# A systematic review and meta-analysis of obesity and COVID-19 outcomes

**DOI:** 10.1038/s41598-021-86694-1

**Published:** 2021-03-30

**Authors:** Xinya Zhang, Alexander M. Lewis, John R. Moley, Jonathan R. Brestoff

**Affiliations:** 1 Department of Pathology and Immunology, Washington University School of Medicine, Mail: 660 S. Euclid Ave - Campus Box 8118, Lab: 425 S. Euclid Ave - 3710 West Building, St. Louis, MO 63110 USA; 2 Skidmore College, Saratoga Springs, NY USA

**Keywords:** Outcomes research, Medical research, Risk factors

## Abstract

Some studies report that obesity is associated with more severe symptoms following SARS-CoV-2 infection and worse COVID-19 outcomes, however many other studies have not reproduced these findings. Therefore, it is uncertain whether obesity is in fact associated with worse COVID-19 outcomes compared to non-obese individuals. We conducted a systematic search of PubMed (including MEDLINE) and Google Scholar on May 18, 2020 to identify published studies on COVID-19 outcomes in non-obese and obese patients, covering studies published during the first 6 months of the pandemic. Meta-analyses with random effects modeling was used to determine unadjusted odds ratios (OR) and 95% confidence intervals (CI) for various COVID-19 outcomes in obese versus non-obese patients. By quantitative analyses of 22 studies from 7 countries in North America, Europe, and Asia, we found that obesity is associated with an increased likelihood of presenting with more severe COVID-19 symptoms (OR 3.03, 95% CI 1.45–6.28, *P* = 0.003; 4 studies, n = 974), developing acute respiratory distress syndrome (ARDS; OR 2.89, 95% CI 1.14–7.34, *P* = 0.025; 2 studies, n = 96), requiring hospitalization (OR 1.68, 95% CI 1.14–1.59, *P* < 0.001; 4 studies, n = 6611), being admitted to an intensive care unit (ICU; OR 1.35, 95% CI 1.15–1.65, *P* = 0.001; 9 studies, n = 5298), and undergoing invasive mechanical ventilation (IMV; OR 1.76, 95% CI 1.29–2.40, *P* < 0.001; 7 studies, n = 1558) compared to non-obese patients. However, obese patients had similar likelihoods of death from COVID-19 as non-obese patients (OR 0.96, 95% CI 0.74–1.25, *P* = 0.750; 9 studies, n = 20,597). Collectively, these data from the first 6 months of the pandemic suggested that obesity is associated with a more severe COVID-19 disease course but may not be associated with increased mortality.

## Introduction

In the first 6 months of human exposure to Severe Acute Respiratory Syndrome (SARS)-Coronavirus-2 (SARS-CoV-2), which causes Coronavirus Disease 2019 (COVID-19), there were over 9,000,000 confirmed infections and nearly 500,000 deaths worldwide^1^. By the end of 2020, it is estimated that there were more than 81 million confirmed cases of COVID-19 and over 1.7 million deaths^1^. Several risk factors have been associated with developing more severe COVID-19 and increased risk of death, including age over 65 years, type 2 diabetes (T2DM), and cardiovascular disease (CVD)^2–4^. Emerging studies have also suggested that obesity is associated with worse COVID-19 outcomes, including increased rates of hospitalization and intensive care unit (IUC) admission, invasive mechanical ventilation (IMV), and death^5–12^. However, many studies have not reproduced these findings^13–22^. In addition, some studies have reported that patients with severe COVID-19 have similar or slightly increased body mass index (BMI) compared to patients with non-severe disease^23–25^. Therefore, it is unclear whether obesity is in fact associated with adverse COVID-19 outcomes or death.

To investigate this, we performed a systematic search of PubMed (including MEDLINE) and Google Scholar on May 18, 2020 to identify all published studies that report COVID-19 comorbidities or outcomes during the first ~ 6 months of the pandemic. We identified 22 cohort studies from 7 countries that could be included in quantitative meta-analyses for six outcomes in obese versus non-obese patients. Our meta-analyses revealed that obese patients are more likely than non-obese patients to present with severe disease, to develop ARDS, need hospitalization, be admitted to the ICU, or require IMV. Surprisingly, however, obesity was not associated with an increased risk of death from COVID-19. Subgroup analysis by continent suggested that in Europe, obesity might be associated with lower mortality from COVID-19 compared to non-obese patients. Although these meta-analyses provide unadjusted odds ratio (OR) estimates, these data from the first 6 months of the pandemic suggest that obesity is associated with a more severe disease course but does not appear to be linked to increased mortality from COVID-19.

## Results

### Literature search and characteristics of the included studies

**Figure 1:**
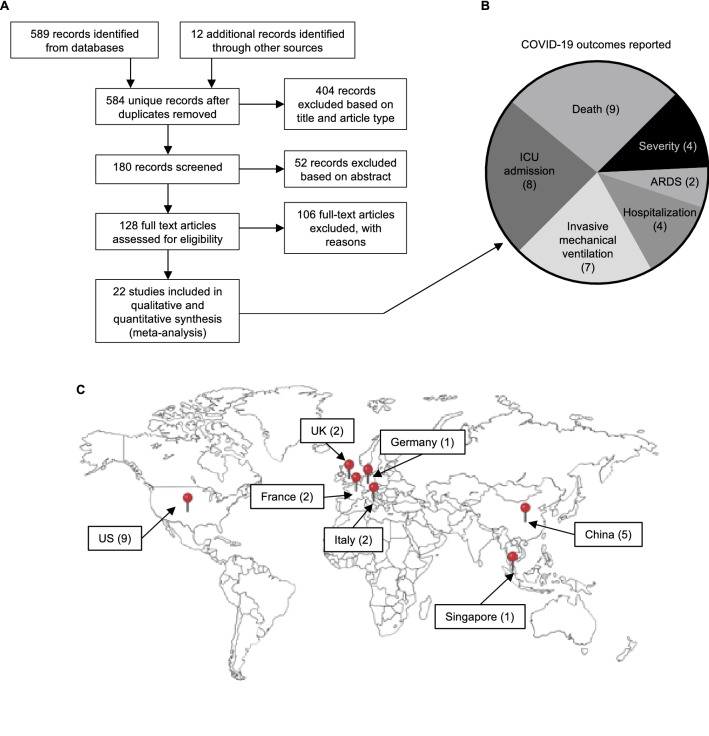
Systematic search strategy and COVID-19 outcomes reported in association with obesity. (**A**) PRISMA Flow Diagram showing the numbers of articles per stage of review, resulting in n = 22 full text articles reporting COVID-19 outcomes stratified on obesity status. (**B**) Six outcomes were reported by the 22 studies included in meta-analysis. The numbers of contributing articles per outcome are in parentheses. Numbers do not add to 22 because some studies report more than one outcome. (**C**) World map showing the locations where the included studies were conducted. The image was generated in BioRender with permission to publish.

Systematic searches of PubMed (including MEDLINE) and Google Scholar returned 589 records that were reviewed by 2 independent investigators (Fig. 1A). There were 12 additional records identified from reviewing the reference lists of full-text articles we assessed for eligibility (see below). This resulted in 584 unique records after duplicates were removed. We excluded 404 records based on manual review of titles for any potential relevance and the article type (e.g. narrative reviews). Of the remaining 180 records, we read the abstracts and excluded 52 records based on no potential relevance. For the remaining 128 studies, we obtained the full text articles which were reviewed in three rounds. In the first round, two independent investigators identified all studies that stratified any COVID-19 comorbidity or outcome on obesity or BMI category. In the second round, a third investigator independently reviewed all of the excluded full text articles to prevent error. In the third round, four authors met in a virtual consensus conference to review all studies with potential for inclusion. For each study that was included, we scanned the reference list which resulted in identification of 12 additional full text articles (128 articles total) that we reviewed in conference. Overall, we excluded 106 full text articles (of 128) because they did not report original COVID-19 outcome data stratified on obesity or BMI category (n = 63), provided insufficient data to include in the meta-analysis (n = 29), or were not a cohort study (n = 14).

This resulted in a total of 22 articles that were included in the meta-analysis (Fig. 1A)^5,6,8–22,26–30^. There were 6 outcomes that were reported by at least 2 of these 22 studies: severity (4 studies representing n = 974 patients), acute respiratory distress syndrome (ARDS, 2 studies representing n = 96 patients), hospitalization (4 studies representing n = 6611 patients), invasive mechanical ventilation (IMV, 7 studies representing n = 1558 patients), being admitted to an intensive care unit (ICU, 8 studies representing n = 5298 patients), and death (9 studies representing n = 20,597 patients) (Fig. 1B). Geographically, these studies were distributed across 3 continents (Fig. 1C). Nine studies were from North America (United States only), 7 studies were from Europe (2 Italy, 2 France, 2 United Kingdom, and 1 Germany), and 6 studies were from Asia (5 China and 1 Singapore). The characteristics of these 22 studies are summarized in Table 1. Study quality was assessed using the Newcastle–Ottawa Scale (NOS) for assessing the quality of nonrandomized studies in meta-analyses, the results of which are provided in Supplemental Table S1. This scoring system assigns stars (up to 9 maximum) for meeting specific quality metrics relating to cohort selection, comparability, and the outcome, where more stars indicates higher quality. The majority of the studies achieved 6 or 7 stars. Four studies received 5 stars, and two studies received 8 stars.

**Table 1:** Study characteristics.

Author, year	Location	Study type	n	Incl. (n)	Excluded (n)	Excluded reason	Ages included (y)	Age (y)	Obesity (kg/m^2^)	Extractable outcome (s)	Sex	Ethnicity	Study setting	SES	Pre-existing conditions	Meds
Busetto et al.^13^	Padova, Italy	RC	92	92	0	n/a	40–96	Mean 70.5 (SD 13.3)	BMI ≥ 30	IMV, ICU, D	Male and female	NR	W	NR	DM2 (30.4%), HF (31.5%)	NR
Cai et al.^14^	Shenzhen, Guangdong China	RC	383	383	0	n/a	≥ 18	NR	BMI ≥ 28	IMV, ICU, S, D	Male and female	Asian	W	NR	NR	NR
Caussy et al.^6^	Lyon, France	RC	357	340	17	BMI unknown	≥ 18	NR	BMI ≥ 30	ICU	NR	NR	W, ICU	NR	DM2 (21%), HF (26%)	NR
Chao et al.^15^	New York, NY, USA	RC	47	47	0	n/a	< 21	Median 13.1 (IQR 0.4–19.3)	BMI ≥ 30	IMV, ICU, ARDS, D	Male and female	White 1 (3%), Black 3 (9.1%), Latino 26 (78.8%), Other 3 (9.1%)	OP, W, ICU	NR	NR	NR
Docherty et al.^20^	United Kingdom	PC	20,133	11,222	8911	4052 BMI unknown; 4859 still receiving care	0–104	Median 73 (IQR 58–82)	"clinician-defined"	D	Male and female	NR	W	NR	DM2 (20.7%)	NR
Dreher et al.^26^	Aachen, Germany	RC	50	50	0	n/a	NR	Median 65 (IQR 58–76)	BMI ≥ 30	ARDS	Male and female	NR	W, ICU	NR	DM2 (58%)	NR
Goyal et al.^10^	New York, NY, USA	RC	393	380	13	BMI unknown	≥ 18	Median 62.2 (IQR 48.6–73.7)	BMI ≥ 30	IMV	Male and female	White 147 (37.4%), Non-white 246 (62.6%)	W	NR	DM2 (25.2%)	NR
Hu et al.^27^	Wuhan, China	RC	323	294	29	BMI Unknown	23–91	Median 62	BMI ≥ 30	S	Male and female	Asian	W	NR	NR	AVT, Abx, CS
Huang et al.^28^	Jiansu, China	RC	202	172	30	BMI unknown	NR	Median 44 (IQR 33–54)	BMI ≥ 28	S	Male and female	Asian	W	NR	NR	NR
ICNARC ^29^	United Kingdom	RC	9347	7521	1826	709 BMI unknown; 1117 still receiving care	≥ 16	Median 60 (IQR 51–68)	BMI ≥ 30	D	Male and female	White 5690 (67%), Mixed 145 (1.7%), 1275 (15%), Black (9.8%), Other 555(6.5%)	W	NR	NR	NR
Kalligeros et al.^16^	Providence and Newport, RI, USA	RC	103	103	0	n/a	≥ 18	Median 60 (52–70)	BMI ≥ 30	ICU	Male and female	White 42 (40.7%), Black 24 (23.3%), Hispanic (35 (33.9%), Asian 2 (1.9%)	W, ICU	NR	DM2 (36.8%),HF (24.2%)	NR
Killerby et al.^12^	Atlanta, GA, USA	RC	531	436	95	BMI unknown	≥ 18	Median 61 (no IQR shown)	BMI ≥ 30	H	Male and female	White 119 (22.4%), Black 313 (58.9%), Other 17 (3.3%), Unknown 82 (15.4%)	OP, W	NR	DM2 (25.5%)	NR
Lighter et al.^9^	New York, NY, USA	RC	3615	1762	0	n/a	NR	NR	BMI ≥ 30	ICU	NR	NR	W	NR	NR	NR
Moriconi et al.^21^	Pisa, Italy	RC	100	100	0	n/a	NR	NR	BMI ≥ 30	D	Male and female	NR	W	NR	NR	NR
Ong et al.^17^	Tan Tock Seng, Singapore	RC	182	91	91	BMI unknown	NR	NR	BMI ≥ 25	IMV, ICU, D	Male and female	Asian	W	NR	NR	NR
Peng et al.^11^	Wuhan, Hubei, China	RC	112	112	0	BMI unknown	≥ 18	Median 62 (IQR 55–67)	BMI ≥ 25	D	Male and female	Asian	W	NR	CVD	ACEI/ARB
Petrilli et al.^5^	New York, NY, USA	RC	5279	5040	239	BMI unknown	≥ 19	Median 54 (IQR 38–66)	BMI ≥ 30	H	Male and female	White 2003 (37.9%), African American 835 (15.8%), Asian 383 (7.3%), Hispanic 1330 (25.2%), Other 397 (7.5%), Unknown 331 (6.3%)	OP, W	NR	DM2 (22.6%)	NR
Rosenberg et al.^22^	New York, NY, USA	RC	1438	1030	408	BMI unknown	NR	Median 63	BMI ≥ 30	D	Male and female	White 167 (24.1%), Black 199 (28.7%), Hispanic 199 (28.7%), Other 128 (18.5%)	W	NR	DM2 (48.9%)	HC or Z
Simonnet et al.^8^	Lille, France	RC	124	124	0	n/a	NR	Median 60 (IQR 51–70)	BMI ≥ 30	IMV	Male and female	NR	ICU	NR	DM2 (23%)	NR
Suleyman et al.^18^	Detroit, MI, USA	RC	463	463	0	n/a	NR	Mean 57.5 (SD 16.8)	Assumed BMI ≥ 30 given study location	H, ICU	Male and female	African American 334 (72.1%), Non-African American 129 (27.9%)	W, ICU	NR	DM2 (38.4%)	Abx
Toussie et al.^19^	New York, NY, USA	RC	338	313	25	BMI unknown	21–50	Median 39 (IQR 31–45)	BMI ≥ 30	IMV, H	Male and female	White 71 (21%), Asian 30 (9%), Black 78 (23%), Hispanic116 (34%), Unknown 43 (13%)	OP, W	NR	NR	NR
Zheng et al.^30^	Wenzhou, China	RC	66	66	0	n/a	18–75	Mean 47 (no SD shown)	BMI ≥ 25	S	Male and female	Asian	W	NR	DM2 (24.2%)	COVID-19 Management guidance (7^th^ edition)

RC, retrospective cohort; PC, prospective cohort; n/a, not applicable; Incl., included; BMI, body mass index; y, years; NR, not reported; IQR, interquartile range; SD, standard deviation; S, severity; ARDS, acute respiratory distress syndrome; H, hospitalization; W, ward; OP, outpatient; ICU, intensive care unit admission; IMV, invasive mechanical ventilation; D, death; N, none; HF, heart failure; DM2, type 2 diabetes mellitus; CVD, cardiovascular disease; Meds, medications; Abx, antibiotics; AVT, antiviral therapy; HC, hydroxychloroquine; Z, azithromycin; CS, corticosteroids; ACEi, angiotensin converting enzyme II inhibitor; ARB, angiotensin II receptor blocker.

### Obesity is associated with a more severe COVID-19 disease course

**Figure 2:**
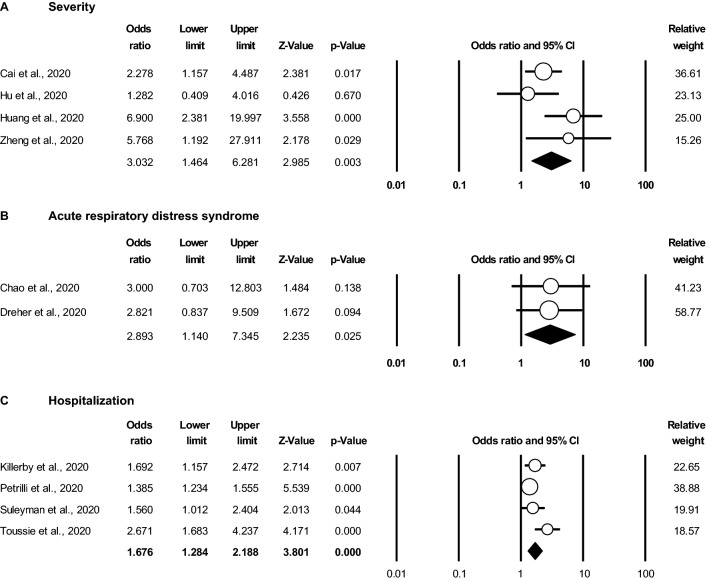
Obesity is associated with more severe COVID-19 and an increased likelihood of acute respiratory distress syndrome (ARDS) and hospitalization. Random effects meta-analyses of odds ratios (OR) and 95% confidence intervals (CI) in obese versus non-obese patients for (**A**) presenting with severe COVID-19 disease (4 studies, n = 915 patients), (**B**) developing acute respiratory distress syndrome (ARDS) (2 studies, n = 96 patients), and (**C**) being hospitalized (4 studies, n = 6,252 patients). Non-obese is defined as the reference group.

To investigate the association between obesity and COVID-19 severity, we performed meta-analyses on each outcome variable. Of the four studies that reported non-severe versus severe disease, three reported a statistically significant increase in disease severity in obese patients compared to non-obese patients with ORs ranging from 1.28 to 6.90^14,28,30^, whereas one study did not report a statistically significant difference^27^. On average, random effects meta-analysis modeling indicated an overall OR 3.03 (95% CI 1.46–6.28, *P* = 0.003), indicating that the 4 studies collectively support an association between obesity and exhibiting more severe COVID-19 disease (Fig. 2A).

Consistent with this observation, there were 2 studies that compared the likelihood of developing ARDS in obese versus non-obese patients^15,26^. Although both studies reported a trend towards an increased likelihood of developing ARDS, neither study alone was statistically significant, likely due to being underpowered. When combined in a meta-analysis with random effects modeling, we found that there was a statistically significant association between obesity and developing ARDS (OR 2.89, 95% CI 1.14–7.35, *P* = 0.025) (Fig. 2B). This finding also supports a relationship between obesity and more severe COVID-19.

From a clinical practice perspective, patients with severe respiratory disease are the most likely to be admitted to the hospital for acute or intensive care. We identified 4 studies that reported on hospitalization rates in obese versus non-obese patients^5,12,18,19^. Each of these studies reported a statistically significant increase in the likelihood of hospitalization for obese patients with COVID-19, with ORs ranging from 1.39 to 2.67. In meta-analysis with random effects modeling, the overall OR for hospitalization in obese versus non-obese patients was 1.68 (95% CI 1.28–2.19, *P* < 0.001) (Fig. 2C).

**Figure 3:**
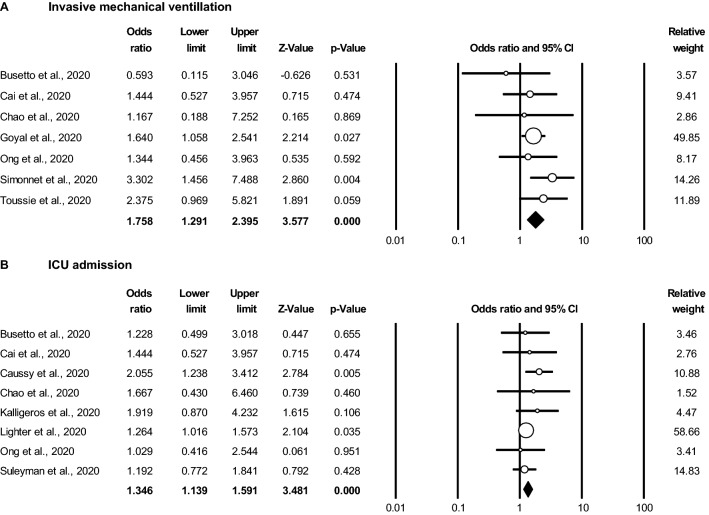
Obesity is associated with an increased likelihood of requiring invasive mechanical ventilation (IMV) and admission to the intensive care unit (ICU) in the setting of COVID-19. Random effects meta-analyses of odds ratios (OR) and 95% confidence intervals (CI) in obese versus non-obese patients for (**A**) requiring IMV (7 studies, n = 1,261) and (**B**) admission to an ICU (9 studies, n = 3227). Non-obese is defined as the reference group.

A subset of COVID-19 patients develop severe respiratory failure that necessitates invasive mechanical ventilation (IMV). There were seven studies that reported IMV in obese and non-obese patients with COVID-19. Of these, two reported a significant increase in the likelihood of IMV in obese versus non-obese patients^8,10^, and a third reported a similar observation with a non-significant *P* value^19^. The other four studies did not report a statistically significant association between obesity and IMV^13–15,17^. Collectively, however, random effects meta-analysis revealed an overall significant increase in the likelihood of IMV in obese versus non-obese patients with an OR 1.76 (95% CI 1.29–2.40, *P* < 0.001) (Fig. 3A).

There were 8 studies that reported ICU admission rates stratified by obesity. Two of these studies reported a statistically significant increase in the likelihood of ICU admission in obese versus non-obese patients^6,9^. The other six studies did not report a statistically significant increase in ICU admission rates in obese patients^13–18^, however all of them reported OR > 1.0 and 5 were likely underpowered to detect a significant difference. When analyzed together in meta-analysis with random effects modeling, overall there was a statistically significant increase in the likelihood of ICU admission in obese versus non-obese patients with an OR 1.35 (95% CI 1.14–1.59, *P* < 0.001) (Fig. 3B).

### Obesity does not appear to be associated with increased COVID-19-associated mortality

**Figure 4:**
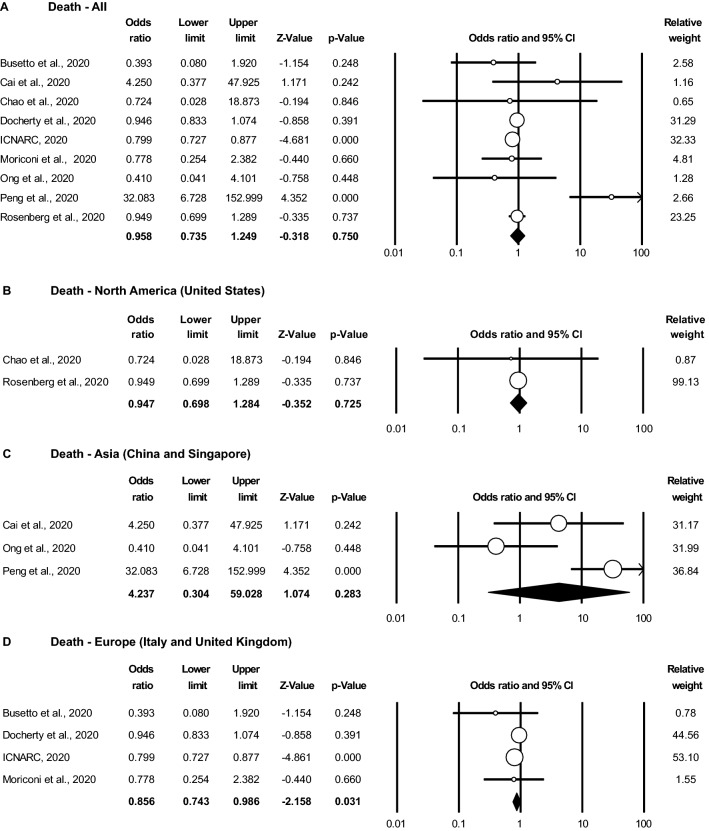
Obesity is not associated with increased COVID-19-associated mortality. Random effects meta-analysis of odds ratios (OR) and 95% confidence intervals (CI) for the likelihood of death in obese vs non-obese COVID-19 patients (**A**) overall (9 studies, n = 20,597 patients and in studies from (**B**) North America (2 studies, n = 1,076), (**C**) Asia (3 studies, n = 586), and (**D**) Europe (4 studies, n = 18,935). Non-obese is defined as the reference group.

There were 9 studies that reported obesity-stratified mortality rates. One relatively small study reported a statistically significant increase in the likelihood of mortality in obese patients with cardiovascular disease ^11^. In contrast, one large study of ICU patients reported a significant decrease in the death rates in obese patients compared to non-obese patients^29^. The other 7 studies did not report a statistically significant unadjusted likelihood of death in obese versus non-obese patients, with OR ranging from 0.39 to 4.25^13–15,17,20–22^. Surprisingly, in meta-analysis with random effects modeling, there was no statistically significant association between obesity and the likelihood of death from COVID-19 with an overall OR 0.96 (95% CI 0.74–1.25, *P* = 0.750) (Fig. 4A).

To assess whether there was a geographic difference in obesity-associated death rates from COVID-19, we performed a subgroup analysis by continent. Two studies from North America (United States) did not show an association between obesity and mortality with an OR 0.95 (95% CI 0.70–1.28, *P* = 0.725) (Fig. 4B). A similar finding was observed in the Asian studies (China and Singapore), where the OR for death in obese versus non-obese patients was 4.24 (95% CI 0.30–59.03, *P* = 0.283) (Fig. 4C). In contrast, in the European studies (United Kingdom and Italy), obesity was associated with a significantly lower likelihood of death, with an OR 0.86 (95% CI 0.74–0.99, *P* = 0.031) (Fig. 4D).

It is known that COVID-19 can result in death over a period of several weeks and that recovery can take up to 6–8 weeks or longer^31^. We hypothesized that the sampling interval for the studies might relate to death rates. To assess this, we calculated the sampling interval for each study as the number of days between the start of enrollment and the end of follow-up. One study^17^ did not report sufficient information to determine the sampling interval and therefore was not included in this analysis. The sampling interval ranged from 20 to 89 days with a mean ± standard error of the mean of 50.3 ± 10.3 days and a median (interquartile range) of 36 (27.75–85.0) days. We did not observe a statistically significant relationship between the sampling interval and the OR for death (Supplemental Figure S1).

### Sensitivity analyses

To determine whether the overall OR and 95% CI were driven by any individual study, we performed remove-one sensitivity analyses for each outcome, in which each study is removed from the analysis and a new overall OR (95% CI) is computed. For COVID-19 disease severity (Supplemental Figure S2A), hospitalization (Supplemental Figure S2B), ICU admission (Supplemental Figure S2C), and IMV (Supplemental Figure S2D), removing any one study had no appreciable effect on the OR effect size or *P* value of the overall association. Remove-one analyses were not computed for ARDS because there were only two studies contributing to that analysis. For death (Supplemental Figure S2E), removing any one study also had no effect on the OR (95% CI), except for one study by Peng et al. Removing this study from the dataset resulted in a statistically significant decrease in mortality in obese patients compared to non-obese patients, with a remove-one OR 0.86 (95% CI 0.78–0.95, *P* = 0.003).

### Publication bias

We performed publication bias analyses and, for each outcome, did not find evidence of publication bias (Supplemental Figure S3). Fail-safe N tests were performed to determine the number of additional studies with OR = 0 that would be needed to abolish statistical significance for each outcome’s meta-analysis (Supplemental Table S2). For ARDS, the fail-safe N statistic was not calculable. For death, we did not observe a statistically significant effect, therefore the fail-safe N was 0. For the other outcomes, the fail-safe N was greater than the number of studies observed in the meta-analyses. We acknowledge that the fail-safe N test has significant limitations, including but not limited to its focus on *P* values and its lack of consideration of study characteristics such as study size, quality, and design. Though publication bias cannot be formally ruled out, both funnel plot and fail-safe N test analyses do not support evidence of publication bias in this meta-dataset.

## Discussion

Using a systemic search strategy and subsequent meta-analysis of 22 studies representing n = 30,141 patients from 7 countries, we show that obesity is significantly associated with several adverse comorbidities and outcomes from COVID-19. Specifically, obese patients are approximately 3-times more likely to present with severe disease or develop ARDS, 1.7 times more likely to be hospitalized, 1.3 times more likely to be admitted to the ICU, and 1.7 times more likely to require IMV. Despite this more severe disease course, obesity was not associated with increased mortality from COVID-19. These data indicate that although obesity is associated with more severe COVID-19 and disease progression, paradoxically these associations do not appear to result in an increased risk of death.

It is not yet known why obese patients develop more severe COVID-19. One possible explanation is that obese patients express higher levels of viral entry factors. Supporting this idea are studies indicating that obesity is associated with increased expression of ACE2, a receptor for SARS-CoV-2 that is required for viral entry^32^, in the human lung bronchial epithelium and other potentially relevant organs such as pericardial adipose tissue^33,34^. These observations suggest that obese patients may have increased susceptibility to SARS-CoV-2 infection. Another possible explanation is that obese patients exhibit chronic pulmonary inflammation, which has previously been linked to an increased risk of developing inflammatory lung diseases and more severe viral pneumonia^35–38^. A third reason that obese patients might exhibit more severe COVID-19 is the effect of obesity on pulmonary mechanics and lung function. Obesity leads to a heavier chest wall that promotes hypoventilation and has been shown to decrease lung compliance and increase lung resistance^39–41^. These physiological parameters may increase the susceptibility of obese patients to develop respiratory failure in the setting of SARS-CoV-2 infection. Further research is needed to investigate these possibilities.

It is not clear why obesity is associated with more severe COVID-19 without being linked to an increase in mortality in our meta-analyses. Our data are restricted to the first 6 months of the pandemic and therefore represent only the first datasets published on mortality in obese and non-obese patients. Subsequently, there have been several large cohort studies^42–44^ that have identified an increased risk in mortality in obese patients infected with SARS-CoV-2, and these studies and likely other relevant studies were not included in our meta-analyses because of the cut-off date for our search. In addition, there are several meta-analyses with later search dates than ours which do show an increased risk of mortality in obese COVID-19 patients. For example, Noor et al. (2020) includes 58 studies that were published since the beginning of the pandemic and showed a pooled risk ratio of 2.18 (95% confidence interval 1.10–4.34) for death in obese vs non-obese patients^45^. Therefore, it is possible that the smaller number of studies in our meta-analysis and time frame of included studies led to type II error. However, if type II error did not occur in our meta-analyses and the association we report is correct, this raises the question of why obese patients do not have increased mortality despite more severe COVID-19. Obesity was associated with more severe disease but improved survival for viral pneumonias (not COVID-19)^46^, a phenomenon known as the obesity paradox, and there is little known about this phenomenon.

An important limitation of our study is that the analyses were not adjusted for other variables that may be related to obesity and COVID-19. Most of the available studies provided raw data on outcomes in obese vs non-obese groups but did not provide results of multivariate (i.e., adjusted) analyses. Some studies did perform multivariate analyses to adjust for age, sex, ethnicity, and/or other variables, however variations in how these studies were performed and the outcomes they reported precluded us from conducting meta-analyses with multivariate models. A second limitation is that BMI category could not be taken into account in our meta-analyses. Most studies did not stratify COVID-19 outcome data by BMI category or reported different BMI category definitions that precluded meta-analysis. It is possible that higher BMI categories (e.g. BMI > 45 kg/m^2^) are associated with increased or decreased risks of some outcomes compared to lower BMI categories (BMI 30–34.9 kg/m^2^). Third, the search date for this systematic review and meta-analysis was inclusive of studies published within the first ~ 6 months of the COVID-19 pandemic, some of which were relatively small or underpowered. It is possible that subsequent larger studies that enable careful adjustment for potential confounding variables could shift the landscape of published literature and could influence the presence and/or strength of associations reported in future meta-analyses.

In conclusion, our systematic review and meta-analyses revealed associations between obesity and COVID-19 comorbidities and outcomes. Obesity appears to be associated with an increased likelihood of having more severe COVID-19, developing ARDS, being hospitalized, being admitted to the ICU, and requiring IMV. However, obesity does not appear to be associated with increased mortality from COVID-19, at least in unadjusted analyses of data published during the first 6 months of the pandemic. These results have direct implications for clinicians because body mass index and obesity status are readily obtainable data that can be used to identify patients at higher risk of a more severe COVID-19 disease course.

## Methods

### Search strategy and exclusion and inclusion criteria

This systematic review and meta-analysis followed PRISMA and MOOSE Guidelines^47,48^. Three authors independently searched PubMed, MEDLINE (through PubMed), and Google Scholar on May 18, 2020 using the following Boolean search terms in the text field:

("COVID-19" OR COVID19 OR "SARS-CoV-2" OR "Severe acute respiratory syndrome coronavirus 2" OR "novel coronavirus" OR nCoV OR “coronavirus disease” or 2019-nCoV OR coronavirus) AND (obesity OR BMI OR "body mass index" OR diabetes OR "metabolic disease" OR "metabolic syndrome")

The search did not include a language, date, or other restriction. The search results from the two databases were merged and duplicates removed. Studies published in or prior to 2018 were then excluded because they could not theoretically directly relate to SARS-CoV-2 or COVID-19. The titles and article types of each remaining entry were manually reviewed by two investigators independently. Review articles, opinions, commentaries, and case reports were eliminated. For all remaining entries, two independent reviewers read all of the abstracts and excluded articles that did not mention obesity, BMI, a metabolic disease or that did not present any new data. Entries that did not include an abstract (e.g., Letter to the Editor) were not eliminated. Abstracts that were not in English were translated by a native speaker if in Chinese or using Google Translate if in another language. After screening titles and abstracts, n = 116 entries remained, and we obtained the full text for each. Two reviewers independently evaluated each full text article to determine whether it reported any COVID-19 outcome or comorbidity stratified by obesity status or BMI category, and these articles were carried forward to the next stage of review. As an additional check, articles that were not carried forward and their reference lists were reviewed by a third independent investigator, who could carry forward any article for further review. In total, n = 128 full text articles were reviewed in a virtual conference call with screen sharing involving all investigators. The methods sections of full text articles were reviewed, and all study types other than cohort studies (retrospective or prospective) were excluded. Any disagreement was resolved by discussion and consensus among all authors. To identify any missed papers, we reviewed the reference lists for each study that was included in the quantitative synthesis. In total, n = 22 articles met inclusion criteria, and from these the reported COVID-19 outcomes were tallied. If two articles reported the same outcome, meta-analysis was performed.

### Definition of obesity

The definition of obesity was BMI ≥ 30 kg/m^2^ for all studies, except for those from Asia, where obesity is typically defined based on lower BMI cutoffs^49^. Definitions of obesity for each study are listed in Table 1.

### Outcomes

The following outcomes were reported by at least two studies: severity of COVID-19 as defined by Diagnosis and Treatment Protocol for Novel Coronavirus Pneumonia (Trial Version 5 or 7)^50^; ARDS as defined by the Berlin Definition criteria^51^; admission to the hospital; admission to an IC; requirement for IMV; or death. For death, the sampling interval was defined as the start of enrollment to the end of the study’s follow-up period.

### Study quality assessment and data extraction

Each of the n = 22 included articles were presented in virtual conference with all authors present. The characteristics and quality of all studies were assessed using the Newcastle–Ottawa Scale (NOS) for assessing the quality of nonrandomized studies in meta-analyses^52^. Data were extracted from each article by one investigator and checked by two other investigators independently. Since adjusted analyses were uncommon and did not use similar covariates, we performed our meta-analyses on unadjusted odds ratios. For several studies^9,10,12,15,17–22,26^, we used data contained with the relevant paper to calculate the numbers of patients in one group or to collapse obese categories into one obese group of BMI ≥ 30 and one non-obese group of BMI < 30 (unless ethnicity-specific BMI cutoffs dictated use of a different cut off for obese vs non-obese, as described above).

### Ethics

This systematic review and meta-analysis includes only anonymized summary data or statistics from previously published studies and therefore is exempt from Institutional Review Board (IRB) review or approval. However, all studies included in this meta-analysis were required to have declared that their relevant IRB or equivalent reviewed and approved the study.

### Statistical analysis

Meta-analysis of each COVID-19 outcome was performed using the Comprehensive Meta-Analysis (CMA) Professional v3 (Engelwood, New Jersey, USA). Odds ratios (OR) and 95% confidence intervals (CI) were calculated by 2 × 2 tables and/or inverse variance approach and analyzed using a random effects model. The Q test was performed to assess the heterogeneity between studies. For meta-analyses with at least three contributing studies, sensitivity analyses were performed by removing one study at a time and comparing the all-included OR and 95% and the various all-minus-one ORs and 95% CIs. Publication bias was assessed in CMA by plotting each outcome variable’s log OR from each study against standard error. Fail-safe N tests were also performed in CMA. Prism 8 was used to relate the sampling interval and mortality OR via linear regression (GraphPad Software, LLC). Statistical significance was set at *P* < 0.05.

## Supplementary Information

Supplementary Information
