# Supplementary material for: A systematic review and meta-analysis of obesity and COVID-19 outcomes
Source: Sci Rep. 2021 Mar 30;11:7193. doi: 10.1038/s41598-021-86694-1 (PMC8009961; doi:10.1038/s41598-021-86694-1)
Supplement: Supplementary file 1 — Supplementary Information [file 41598_2021_86694_MOESM1_ESM.docx]

**SUPPLEMENTAL INFORMATION**

**for**

**A systematic review and meta-analysis of obesity and COVID-19 outcomes**

Xinya Zhang^, Alexander M. Lewis^, John R. Moley^, and Jonathan R. Brestoff

^Contributed equally

**Supplemental Table S1**

**Supplemental Table S2**

**Supplemental Figure S1**

**Supplemental Figure S2**

**Supplemental Figure S3**

**SUPPLEMENTAL TABLES**

**Supplemental Table S1. Scoring of including studies using the Newcastle-Ottawa Scale (NOS) for assessments of quality of non-randomized studies in meta-analyses**

| Author, year | Selection | | | | Comparability | Outcome | | | TOTAL  STARS |
| --- | --- | --- | --- | --- | --- | --- | --- | --- | --- |
|  | 1  Representativeness | 2  Selection of non-exposed | 3  Exposure Ascertainment | 4  Outcome timing | 1  Control | 1  Assessment of outcome | 2  Follow-up long length | 3  Adequacy of follow-up |  |
| Busetto et al., 2020 | **🟑** | **🟑** | **🟑** | IMV: **◯**  ICU: **🟑**  D: **◯** | **◯** | **🟑** | **🟑** | **◯** | IMV: 5  ICU: 6  D: 5 |
| Cai et al., 2020 | **🟑** | **🟑** | **🟑** | IMV:**🟑**  ICU:**🟑**  S: **◯**  D:**🟑** | **🟑** | **🟑** | **🟑** | **🟑** | IMV: 8  ICU: 8  S: 7  D: 8 |
| Caussy et al., 2020 | **🟑** | **🟑** | **🟑** | ICU: **◯** | **🟑** | **🟑** | **🟑** | **◯** | ICU: 6 |
| Chao et al., 2020 | **🟑** | **🟑** | **🟑** | IMV:**🟑**  ICU:**🟑**  ARDS:**🟑**  D:**🟑** | **🟑** | **🟑** | **🟑** | **◯** | IMV: 7  ICU: 7  ARDS: 7  D: 7 |
| Docherty et al., 2020 | **🟑** | **🟑** | **🟑** | D: **◯** | **◯** | **🟑** | **🟑** | **🟑** | D: 6 |
| Dreher et al., 2020 | **🟑** | **🟑** | **🟑** | ARDS: **◯** | **◯** | **🟑** | **🟑** | **◯** | ARDS: 5 |
| Goyal et al., 2020 | **🟑** | **🟑** | **🟑** | IMV:**🟑** | **🟑** | **🟑** | **🟑** | **◯** | IMV: 7 |
| Hu et al., 2020 | **🟑** | **🟑** | **🟑** | S:**🟑** | **◯** | **🟑** | **🟑** | **🟑** | S: 7 |
| Huang et al., 2020 | **🟑** | **🟑** | **🟑** | S: **◯** | **◯** | **🟑** | **🟑** | **◯** | S: 5 |
| ICNARC, 2020 | **🟑** | **🟑** | **🟑** | D:**🟑** | **🟑** | **🟑** | **🟑** | **🟑** | D: 8 |
| Kalligeros et al., 2020 | **🟑** | **🟑** | **🟑** | ICU: **◯** | **🟑** | **🟑** | **🟑** | **◯** | ICU: 6 |
| Killerby et al., 2020 | **🟑** | **🟑** | **🟑** | H:**🟑** | **◯** | **🟑** | **🟑** | **◯** | H: 6 |
| Lighter et al., 2020 | **🟑** | **🟑** | **🟑** | ICU:**🟑** | **◯** | **🟑** | **🟑** | **◯** | ICU: 6 |
| Moriconi et al., 2020 | **🟑** | **🟑** | **🟑** | D:**🟑** | **◯** | **🟑** | **🟑** | **◯** | D: 6 |
| Ong et al., 2020 | **🟑** | **🟑** | **🟑** | IMV: **◯**  ICU: **◯**  D: **🟑** | **◯** | **🟑** | **🟑** | **◯** | IMV: 5  ICU: 5  D: 6 |
| Peng et al., 2020 | **◯** | **🟑** | **🟑** | D: **🟑** | **🟑** | **🟑** | **🟑** | **◯** | D: 6 |
| Petrilli et al., 2020 | **🟑** | **🟑** | **🟑** | H: **◯** | **🟑** | **🟑** | **🟑** | **◯** | H: 6 |
| Rosenberg et al., 2020 | **🟑** | **🟑** | **🟑** | D:**🟑** | **◯** | **🟑** | **🟑** | **🟑** | D: 7 |
| Simonnet et al., 2020 | **🟑** | **🟑** | **🟑** | IMV: **◯** | **◯** | **🟑** | **🟑** | **◯** | IMV: 5 |
| Suleyman et al., 2020 | **🟑** | **🟑** | **🟑** | ICU:**🟑**  H:**🟑** | **◯** | **🟑** | **🟑** | **◯** | ICU: 6  H: 6 |
| Toussie et al., 2020 | **🟑** | **🟑** | **🟑** | IMV:**🟑**  H: **◯** | **🟑** | **🟑** | **🟑** | **◯** | IMV: 7  H: 6 |
| Zheng et al., 2020 | **🟑** | **🟑** | **🟑** | S: **◯** | **🟑** | **🟑** | **🟑** | **◯** | S: 6 |

S, severity; ARDS, acute respiratory distress syndrome; H, hospitalization; ICU, intensive care unit admission; IMV, invasive mechanical ventilation; D, Death; **🟑**

point awarded; **◯**, no point awarded

**Supplemental Table S2. Fail-safe N test results for each outcome in meta-analysis**

| **Outcome** | **Number of observed studies** | **Number of additional studies with OR=0 needed to abolish statistical significance in observed meta-analyses**  **(fail-safe N)** |
| --- | --- | --- |
| ARDS | 2 | NC |
| Death | 9 | 0 |
| Hospitalization | 4 | 51 |
| ICU Admission | 8 | 15 |
| IMV | 7 | 9 |
| Severity | 4 | 16 |

ARDS, acute respiratory distress syndrome; ICU, intensive care unit; IMV, invasive mechanical ventilation; OR, odds ratio; NC, not calculable.

**SUPPLEMENTAL FIGURES AND LEGENDS**

**Supplemental Figure S1. Obesity-associated mortality in COVID-19 patients does not appear to be a function of sampling interval.** The sampling interval (days) for each study was determined and plotted against the OR and 95%CI for that study. One study (Ong et al., 2020) did not report sufficient information to determine the sampling interval and was therefore excluded from this analysis. Linear regression was performed. The best-fit line and P-value are shown, where the P-value is for whether the slope differs from zero.

**Supplemental Figure S2. Remove-one sensitivity analyses for outcomes reported by at least three studies.** The listed study was removed from the dataset, and a new remove-one odds ratio (OR) and 95% confidence interval (95%CI) was computed and compared against the all-in OR and 95%CI. **(A)** COVID-19 severity, **(B)** hospitalization, **(C)** intensive care unit (ICU) admission, **(D)** invasive mechanical ventilation (IMV), and **(E)** death are shown. Remove-one sensitivity analysis was not performed for ARDS because only two studies reported this complication. Remove-one and all-in OR with 95% confidence intervals are shown alongside P-values.

**Supplemental Figure S3. Assessment of publication bias for all outcomes included in this meta-analysis.** The log OR for each contributing study was plotted against standard error for **(A)** COVID-19 severity, **(B)** hospitalization, **(C)** intensive care unit (ICU) admission, **(D)** invasive mechanical ventilation, and **(F)** death. For each graph, the triangle defines where P=0.05.
